# The Improved Catheterization Is Associated With the Deeper Radial Arteries in Ultrasound-Guided Dynamic Needle Tip Positioning Technique

**DOI:** 10.3389/fmed.2022.803124

**Published:** 2022-02-15

**Authors:** Yuan Tian, Bing Bai, Yuelun Zhang, Lu Che, Jin Wang, Yi Wang, Chunhua Yu, Yuguang Huang

**Affiliations:** ^1^Department of Anaesthesiology, Peking Union Medical College Hospital, Chinese Academy of Medical Sciences and Peking Union Medical College, Beijing, China; ^2^Medical Research Center, Peking Union Medical College Hospital, Chinese Academy of Medical Sciences and Peking Union Medical College, Beijing, China

**Keywords:** ultrasonography, radial artery, catheterization, dynamic needle tip positioning, depth

## Abstract

**Objective:**

This study aimed to determine the associations between the first-attempt success and arterial depth in ultrasound-guided radial artery catheterization (RAC) with dynamic needle tip positioning (DNTP) technique. This study also aimed to further explore the cut-off depth correlated to improved first-attempt success catheterization in less time.

**Methods:**

The cases undertaken by RAC within the DNTP technique between March 2019 and July 2020 were extracted from our institutional cohort database. Relevant variables were collected, including patients' demographics and catheterized information. Univariable and multivariable logistic regression analyses were performed to determine the association. The receiver operating characteristic (ROC) curve and the Youden index were used to explore the cut-off values of the arterial depth. Categorized cases according to the cut-off values, the Kaplan-Meier analysis, and the log-rank test were used to determine the difference of first-attempt success with limited catheterized time between groups.

**Results:**

In this study, 119 patients were enrolled and 98 achieved first-attempt success. The first-attempt success catheterization was observed to be correlated to arterial depth (*p* < 0.01, odds ratio 6.47). An optimal cut-off depth of 2.25 mm was found using the Youden index (0.53) by ROC curve (area under curve 0.77). Arterial depth of more than 2.25 mm was correlated to improved first-attempt success catheterization in less time (log-rank *p* < 0.01).

**Conclusion:**

To achieve first-attempt success catheterization using the DNTP technique, an arterial depth of more than 2.25 mm was associated with less catheterized time.

## Introduction

In anesthesia, intensive care, and emergent medicine, radial artery catheterization (RAC) is a significant procedure, that allows real-time blood pressure administration and provides convenient blood sampling when needed. The ultrasound-guided catheterization has been widely accepted for the superiority of enhancing the success rate and reducing requirements for attempts and time (1–5), especially encountered the patients with hemodynamic unstable, obesity, edema, or with the artery of small size, tortuous, or spasm (6, 7). Nevertheless, further improvements remain moving forward to wrestle with complex circumstances and ensure consistency in outcomes.

Among the growing applications of ultrasound-guided techniques, the dynamic needle tip positioning (DNTP) technique was first reported using in RAC in 2012 (8). The DNTP technique is a modified short-axis, out-of-plane, ultrasound-guided technique. It focuses on discriminating the needle tip during artery puncturing until the tip of the catheter be inserted into the center of the artery (8–10). As mentioned in the meta-analysis of 12 randomized control studies enrolled 2,432 adult participants, which suggests ultrasound-guided DNTP technique and long-axis view performed better than conventional short-axis technique in improving first-attempt success (7). Though both the DNTP technique and long-axis view are committed to discriminating the needle tip, using the DNTP technique hardly worry about the time-consumption of vascular imaging (11) and losing target because of thickness artifacts (12).

Notably, the success rates of ultrasound-guided catheterization varied between studies, and the associating factors remained uncertain (13–21). Though few operators identify the arterial depth before catheterization, it was considered as associated with the performance of ultrasound-guided RAC. In pediatric patients, the arterial depth was observed to be associated with the success rates of the ultrasound-guided short-axis RAC (22). The secondary analysis of our previous randomized controlled trial demonstrated the associations of the arterial depth and complications of DNTP or conventional short-axis ultrasound-guided RAC in adults (23). Hence, it hasn't been well-established if the arterial depth is associated with the first-attempt success of DNTP-guided RAC in adults and its optimal cut-off value. Therefore, we retrospective reviewed the records of the DNTP-guided RAC to determine the association of the arterial depth and the first-attempt success, and further explore the cut-off depth associated with improved first-attempt success in less time.

## Methods

### Study Design and Participants

A retrospective cohort study was conducted in Peking Union Medical College Hospital, a tertiary comprehensive hospital. This study was approved by the Institutional Review Board and Ethics Committee (S-K1366, September 11, 2020), and the need for informed consent was waived. Adult patients undergoing elective surgery who required RAC and were fulfilled by a certain anesthesiologist using the DNTP technique during March 2019 and July 2020 were involved. The anesthesiologist who performed all the cases was experienced in radial arterial catheterization and fulfilled more than 50 DNTP-guided catheterizations under supervision.

### Data Collection

Data clinically relative to the first-attempt success catheterization were collected. Data were obtained from the institutional specialized cohort database of recording ultrasound-guided vascular catheterization (Hospital Clinical Research Database; Beijing Huiren Technology Development Co., Ltd., Beijing, China). The cohort database is currently used for recording the clinical practices, which was first built for our previous randomized controlled study that compared the efficiency of different methods for RAC (16).

### Variables and Outcomes

Patient demographics and arterial characteristics were collected, including sex, age, body mass index (BMI), history of coronary artery disease (CAD), systolic blood pressure (SBP), diastolic blood pressure (DBP), American Society of Anesthesiologists (ASA) physical status grade, and arterial diameter and depth. The diameter and depth of the artery were measured twice at the site of puncturing before catheterization, and the mean of measurements was recorded. The diameter was defined as the distance between the anterior and posterior walls of the artery. The depth was defined as the distance between the transducer and the anterior wall of the artery.

The primary outcome is the first-attempt success, defined as successful catheterization at the first attempt without withdrawing the needle out of the skin. The secondary outcome is the catheterized time, calculated between the needle puncturing into the skin and fulfilled the catheterization with no more than five attempts or 10 min.

### Statistical Analysis

As a retrospective study, we estimated the statistical power using the available sample size of 119 patients with 98 first-attempt successes. With mean values of 2.8 and 2.195 of depth in the success and failure groups and an *SD* value of 0.599 (median of 0.68 and 0.517), statistical power was 98.6%.

Variables considered as clinically significant were included in multiple logistic regression analysis with forwarding selection and results were performed as odds ratio (OR), 95% CI, and *p*-value. By using the receiver-operating characteristic (ROC) analysis, the Youden index was calculated to define the cut-off value. Patients were further categorized based on the cut-off value and the Kaplan-Meier curve and log-rank test were used to compare the first-attempt success rates with limited operation time. Statistical analysis was conducted using SPSS version 22 (IBM Corp, IL, USA). A two-sided *P*-value < 0.05 was considered statistically significant.

## Results

Overall, 119 patients were recruited, in which 98 (82.35%) of them were fulfilled at the first attempt. Demographic characteristics and catheterized information are shown in Table 1. The results of binary logistic regression analysis are shown in Table 2. Age, history of CAD, arterial diameter, and depth were independently correlated to the first-attempt success of RAC. After adjustment with age, history of CAD, and arterial diameter, the depth remained significant effect on first-attempt success (OR 6.47, 95% CI 2.16–19.41). The Hosmer-Lemeshow goodness-of-fit statistic (*P* = 0.82) indicated that the model was well-calibrated.

**Table 1:** Demographic characteristics and catheterized information.

**Parameters**	**Values (min-max)**
Age (years)	60.21 (21–82)
Sex (male/female)	71/48
BMI (Kg/m^2^)	23.9 (15.4–36.7)
CAD (yes/no)	58/61
SBP (mmHg)	130 (84–174)
DBP (mmHg)	76 (45–127)
ASA grade (I/II/III/IV)	0/2/117/0
Diameter (mm)	2.49(1.3–3.5)
Depth (mm)	2.69(1.6–4.3)
First-attempt success (yes/no)	98/21
Overall success (yes/no)	115/4
Catheterization time (s)	110 (12–600)

*BMI, body mass index; CAD, coronary artery disease; SBP, systolic blood pressure; DBP, diastolic blood pressure; ASA grade, American Society of Anesthesiologists physical status grade*.

**Table 2:** Factors associated with the first-attempt success of DNTP guided RAC with multiple logistic regression analysis.

**Dependent variable=first-attempt success catheterization or not**			
	**OR**	**95% CI**	***P***
Age (years)	0.94	0.89–1.00	0.03^*^
Sex (male/female)	-	-	0.87
BMI (Kg/m^2^)	-	-	0.75
CAD (yes/no)	3.58	1.04–12.32	0.04^*^
SBP (mmHg)	-	-	0.82
DBP (mmHg)	-	-	0.87
ASA grade (II/III)	-	-	0.92
Diameter (mm)	4.17	1.23–14.17	0.02^*^
Depth (mm)	6.47	2.16–19.41	<0.01^*^

*OR, odds ratio*.

* *Statistically significant*.

**Figure 1:**
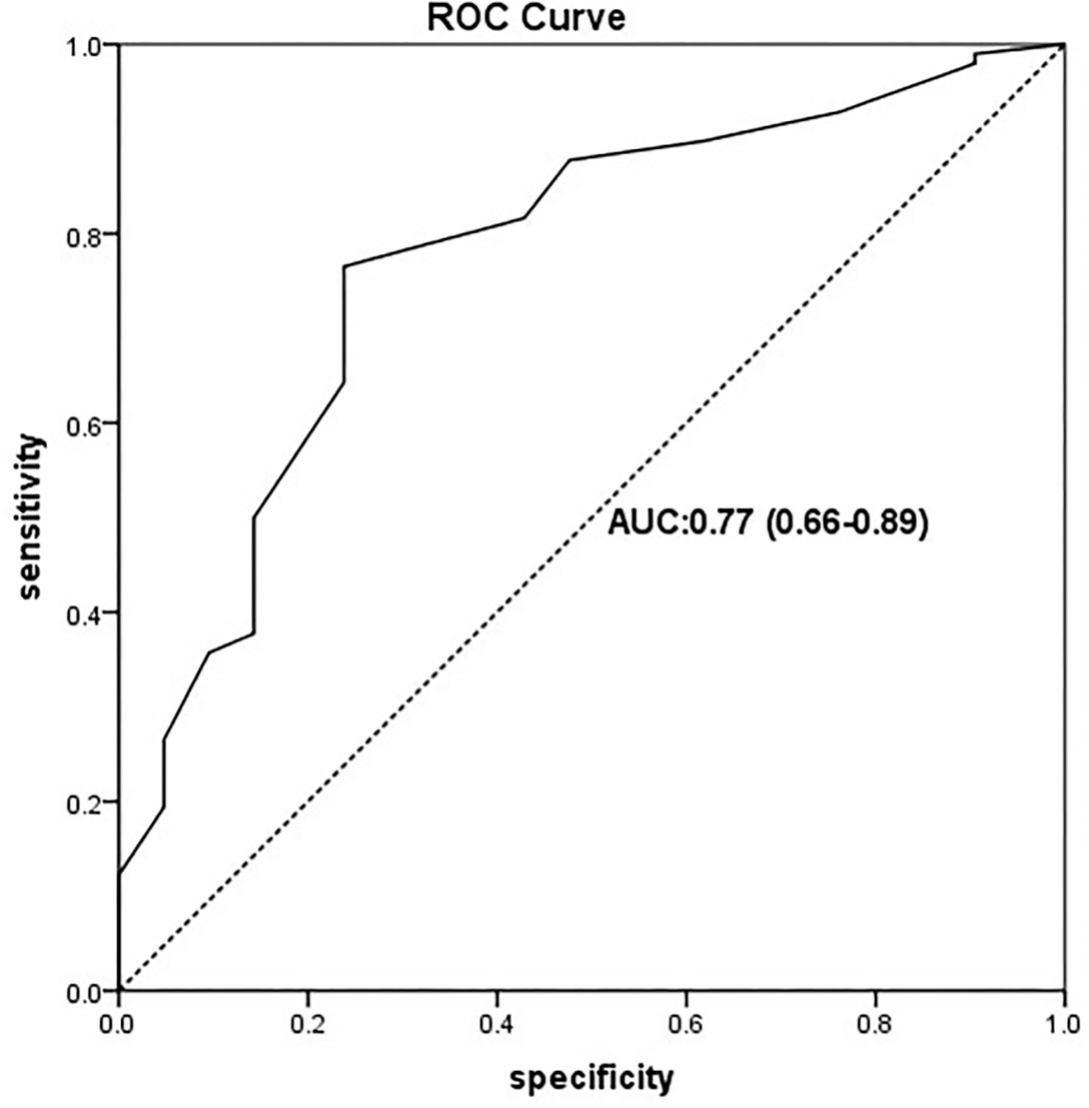
ROC curve of the predictive model for the first-attempt success of radial artery catheterization (RAC) by the arterial depth. ROC curve with an AUC value of 0.77 (95% CI: 0.66–0.89). ROC, receiver-operating characteristic ROC; AUC, area under the ROC curve.

The cut-off value was analyzed by the ROC curve (Figure 1). According to the criterion of maximizing the Youden index (0.53), the optimal cut-off value for first-attempt success has corresponded to the arterial depth of 2.25 mm.

**Figure 2:**
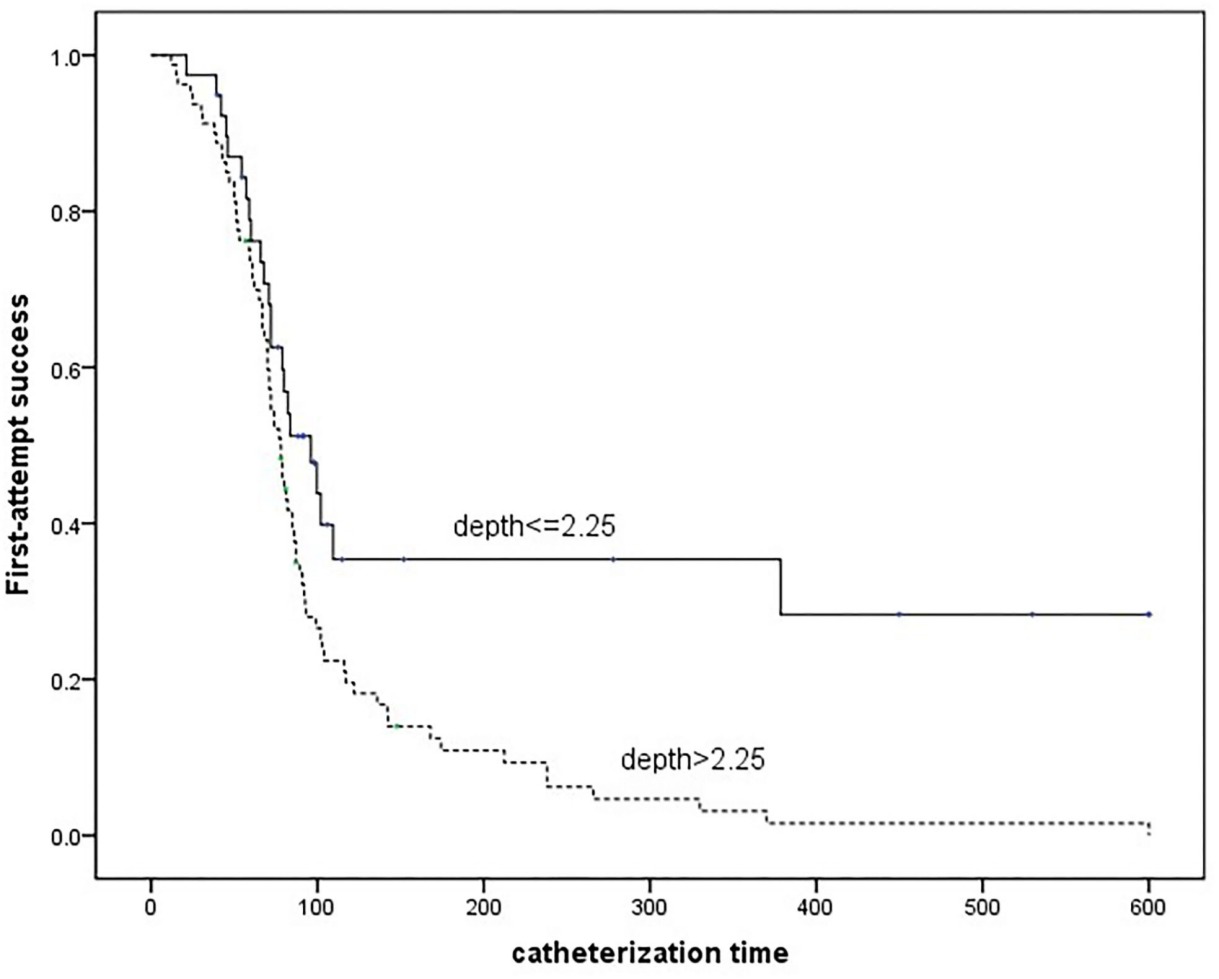
Kaplan-Meier curve estimates the differences of time required for the first-attempt success grouped by the arterial depth.

As shown in Figure 2, to achieve first-attempt success, a significantly shorter catheterized time was required for the deeper artery group compared with the shallow group (log-rank *P* < 0.01).

## Discussion

The current study determined the association between the depth and the success rates of DNTP-guided RAC in adults. The previous study among pediatrics has reported the association between the increased depth and the improved success of catheterization using the conventional ultrasound-guided short-axis technique (22). The reason was that the needle tip can be well-adjusted to puncture into the center when there was enough distance from the anterior arterial wall. In addition, the key to the success of DNTP is identifying the needle tip, hence, it's less likely to distinguish the hyperechoic needle tip from the similar subcutaneous tissues when they are close to each other. These factors probably explained why the depth associated with the catheterization success of the DNTP technique.

According to the current study, it was estimated an optimal depth of more than 2.25 mm was associated with an improved first-attempt success rate of DNTP-guided catheterization. It was demonstrated that the depth of 2–4 mm improved the success rates of conventional short-axis ultrasound-guided RAC in children younger than 3 years old (22). We considered that the difference might be due to differences in methods used for catheterization, as a 95% success rate of DNTP-guided RAC was reported in arteries deeper than 4 mm (18). In patients with a deep artery, the success rates of conventional short-axis ultrasound-guided RAC might be low. The visualization of the needle tip was slashed because a steeper puncture angle was required to achieve the deep artery with constant ultrasonography. The DNTP technique enables positioning the needle tip by moving the transducer, therefore the puncture angle can be adjusted according to the visualization of the ultrasonography instead of the advancement trajectory. On the other hand, to our minds, distinguishing the needle tip in a routine procedure using a catheter with an outer diameter of 1.1 mm is not easy. Because it means that similar hyperechoic images of the arterial anterior wall and the needle tip are required to be distinguished within a distance of 0.6 mm. As the result has not previously been described, further research on the current topic is recommended.

The current study also indicated that to achieve first-attempt catheterization, the deeper arteries required less time. The previous study of the DNTP technique in neonates demonstrated trainees could achieve a higher success rate taking less time by increasing depth with saline injection (21). The reason is supposed, though a deeper artery associated with a potential longer catheterized trajectory, clear identification of needle tip saved the time.

Several limitations of this study should be underlined. First, the same operator, who was sufficiently experienced in ultrasound-guided arterial catheterization of the DNTP technique, performed all the procedures. This limited the external validity of the results. Since the objective of the current study was to determine the association between arterial depth and success catheterization, the different experiences of the operators should be controlled. Interestingly, the consistent results were demonstrated in trainee anesthesiologists that can achieve higher success rates by increasing the arterial depth (21). Second, as a retrospective study, we admitted that confounders other than the finally included factors were not well-controlled in the analysis. Future studies are needed to further investigate this subject.

In conclusion, the current study demonstrated associations between the arterial depth and the first-attempt success of ultrasound-guided RAC using the DNTP technique. An arterial depth of more than 2.25 mm is associated with less time required to achieve first-attempt success.

## Data Availability Statement

The data used in this study are available on request to the corresponding author.

## Ethics Statement

The studies involving human participants were reviewed and approved by the Institutional Review Board and Ethics Committee of Peking Union Medical College Hospital. Written informed consent for participation was not required for this study in accordance with the national legislation and the institutional requirements.

## Author Contributions

YT and BB contributed to the design of the study, administration of the study, data collection, data analysis, and manuscript preparation. YZ contributed to data analysis and critical manuscript review. LC, JW, and YW contributed to the design and data collection. YH contributed to the design of this study and the administration of the study. All authors read and approved the final manuscript. All authors contributed to the article and approved the submitted version.

## Funding

The current research is supported by the CAMS Innovation Fund for Medical Sciences (CIFMS) (2021-I2M-C&T-B-020).

## Conflict of Interest

The authors declare that the research was conducted in the absence of any commercial or financial relationships that could be construed as a potential conflict of interest.

## Publisher's Note

All claims expressed in this article are solely those of the authors and do not necessarily represent those of their affiliated organizations, or those of the publisher, the editors and the reviewers. Any product that may be evaluated in this article, or claim that may be made by its manufacturer, is not guaranteed or endorsed by the publisher.
